# Application of Clustering Algorithm in Corporate Strategy and Risk

**DOI:** 10.1155/2022/8803375

**Published:** 2022-08-31

**Authors:** Qiong Wen

**Affiliations:** Hunan University of Information Technology, Changsha 410151, Hunan, China

## Abstract

With the increasing rise of market mechanism and corporate strategic management theory, many companies, especially listed companies, take diversification as their basic strategy. As the main source of power to promote the rapid development of China's economy, listed companies have also changed the way of China's economic development. It increases the speed of economic development, improves the level of economic technology, and accelerates the process of economic internationalization. Any economic behavior of an enterprise involves risks. As the development plan to mobilize the overall interests of the enterprise, the strategy will definitely involve the long-term development direction of the enterprise. Enterprise strategic deployments to strategic managements are affected by risk control elements. Strategic risk, as an unavoidable practical problem for enterprises, must be managed. Managing strategic risk is the key to effectively evaluate the management and control system. In order to solve this problem, this paper introduces a clustering algorithm, classifies the strategic risk of diversification, and analyzes its causes. For an in-depth analysis of the risks that diversification will face, this paper selects the case of the enterprise's diversification development to carry out the research. It conducts in-depth analysis and research on its basic situation, diversification strategy, dilemma, and behavior. The experimental results show that the financial leverage coefficients of the company in the four quarters are 24.64, 3.93, 104.71, and 6.59 respectively. From the numerical value, it can be concluded that the enterprise is in a high-risk state and the strategic risk status of the company can be accurately judged.

## 1. Introduction

At present, the social and economic development of the world is a trend of globalization, and the competition among many enterprises is getting stronger and stronger. Large enterprises are gradually shifting their development strategies to diversification, thereby increasing their significant market share in the industry. China's strategic risk research started relatively late. With the deepening of reform and opening up, the adjustment of the economic structure, the clarification of the property rights system, and the real integration of the enterprise with the market, it gradually strengthens its risk strategy awareness. Strategic risk management control is to comprehensively grasp the internal and external uncertain factors of the enterprise in combination with some problems of the enterprise itself in the process of implementing the company's strategy. It formulates effective strategic goals and identifies, analyzes, and controls changes in the company's environmental resources and competitiveness factors in the process of strategy implementation, so as to more effectively achieve the company's long-term strategic goals. Therefore, it is very important to carry out strategic risk analysis for enterprises.

Related scientists have done the following research on corporate strategy methods. Haug et al. uses modern portfolio and corporate diversity theories. A comprehensive set of assumptions is developed about the impact of consistent and unrelated fluctuations on a company's systematic risk, overall risk, and risk of failure. It was found that systemic risk was not reduced due to firm diversification, whereas bankruptcy risk was evident in diversified firms [1]. Hashimoto and Ozawa conducted a simulation of a firm's strategy for utilizing directly hired construction workers. The study found that the direct employment strategy resulted in the loss of surplus workers due to the smaller winning contract amount, but avoided labor shortage and skill decline [2]. The purpose of St. Hilaire, WA, is to assess the need to include a provision for credit losses and risk measures to assess the impact on prepayment and profitability models. The effect of the settlement variables on the bank's profitability and on the advance payment methods is examined. Credit risk proved to be more important for Nigerian banks than for South African banks [3]. Vormedal examines the role of companies in promoting sustainable aquaculture and regulation with an emphasis on the world's largest salmon producer. Explore why a leading multinational chooses an aggressive sustainability policy. A new model of pluralism has been developed to understand the driving forces behind the development of active methods [4]. Yuan et al. examines the relationship between a company's corporate policy and corporate social responsibility. A comprehensive measurement of enterprise policy with a theoretical framework shows that companies with an innovation-oriented policy have better social responsibility than companies with an efficiency policy [5]. The flaw in the current approach to corporate strategy lies in the inaccurate prediction of risk.

Regarding clustering algorithms, relevant scientists have done the following research. Zafar and Mahmood introduced a system for determining the efficiency of cluster algorithms in wireless sensor networks. The wireless sensor network is a rapidly evolving technology. Such networks have the ability to share tasks among themselves to perform efficient computations, and some suggestions have been made that can help improve the efficiency of cluster algorithms [6]. Sangaiah et al. proposed three methods: uncontrolled, semicontrolled methods, and dimensional subtraction techniques to create cluster-related classifications for Arabic text documents. Compared to other methods, these methods show better accuracy and fewer errors in the new classification test cases [7]. Yoganathan et al. proposed a new data-based approach based on field measurements in office buildings to obtain the best measurement points (number and location). The cluster algorithm, the data loss method, and the Pareto principle are used to find the best method for the sensor location. The results of this research will have significant implications for researchers and experts [8]. Xue and Wang developed a novel density clustering method for incomplete data based on Bayesian theory. The method performs attribution and clustering simultaneously and utilizes intermediate clustering results. The experimental results show the effectiveness of the proposed algorithm [9]. Mittal performed a comprehensive protocol analysis for low-power adaptation mutation clusters of wireless sensor networks. The proposed maximum clustering system is extensively reviewed and the optimized clustering methods are compared based on various performance variables. Central cluster methods based on dynamic concepts are more suitable for applications requiring low energy, high data transfer rate, or high flexibility than algorithms based on other ideologies [10]. Shang et al. proposed a new exponential system and compositional properties of differential expression. Different identities were applied to the two cluster algorithms in the data frame of the design. The experiments show that both cluster algorithms satisfy the differential analysis [11]. Ezugwu et al. presented a systematic review of the class and a bibliographic analysis of the flows and evolution of the methods of nature-inspired clusters. The study also deals with the main problems of meteorology such as design and the main area of application of cluster problems [12]. Bipul Hossen and Rabiul Auwul conducted a comparative study of the four most popular cluster masks. He also analyzes eight real genetic cancer data and simulated databases. Based on the comparative results of seven popular cluster validity indicators, this study provides a practical analytical framework for obtaining cluster results from data-based cancer gene expression [13, 14]. Mahesh Prabhu et al. collected data related to the appropriate attributes of supplier logistics and proposed a method for optimizing supplier logistics using clustering algorithms. Experiments are conducted on supplier logistics data from different countries, and the results of this work can help buyers select cost-effective suppliers that meet their business requirements [15]. Sami et al. conducted a comparative study of biclustering algorithms. These algorithms are used in numerical experiments to evaluate the large size of submatrices found in the original matrix. In the given two datasets, two algorithms that have gained attention in the field of biclustering in recent years are discussed and tested [16]. The above studies analyze in detail the application of cluster algorithms and methods to a company's strategy. There is no doubt that these studies have made a significant contribution to the development of similar fields. It can teach many techniques and data analysis. However, the clustering algorithms of business policy methods have been relatively little studied and it is necessary to fully apply these algorithms to research in this field [17].

It lists all the combinations of independent programs and selects the project group whose investment amount is not greater than the capital budget. The company's net profit growth rate and the industry's average net profit growth rate are obtained. The net profit growth rates in 2015, 2016, and 2017 were 57.06%, 87.5%, and 48.01%, respectively. The average net profit growth rate of the same industry was 14.43%, 11.69%, and 11.16%, respectively. The comprehensive risk analysis of the company was carried out, and the financial leverage coefficients were obtained, which were 24.64, 3.93, 104.71, and 6.59, respectively. It can be seen that the company's financial leverage has not fluctuated much in recent years.

## 2. Method

### 2.1. Corporate Strategy and Risk Approach

**Figure 1:**
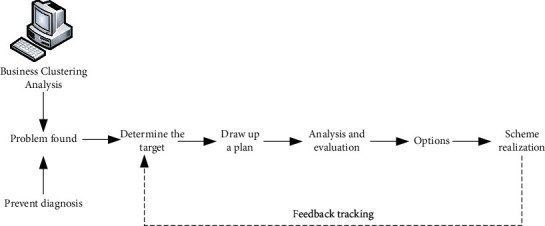
Basic program diagram.

In this paper, a clustering algorithm is introduced to conduct cluster analysis on the number of words related to the company's strategic risk, so as to carry out the research on the company's strategy and risk. The basic program diagram is shown in Figure 1.

Enterprise risk management and control play a significant role in the actual operation of an enterprise. A smooth landing, good operations, continuous coordination, and market compatibility are the results that management most wants to see. Risk management and control are mixed together and have formed an inseparable whole, so it has the following characteristics. The important function of risk management and control lies in the prediction and analysis of the enterprise's work. Through practical activities to recognize risk management and control, the extensive insurance awareness will be transformed into a risk management and control method. This is the value of risk management. How the governance of the main body of the enterprise environment looks at the risk and starts to control the risk, and sets the risk management and control in the real environment of the enterprise, and establishes the enterprise's own moral system and value orientation to adapt to the changes of the external environment. Enterprise risk management and control are to control the information of the enterprise's risk factors and understand the communication at all levels of the enterprise. Only by understanding the practical significance of risk management and control can risk assessment and risk response be made.

The company's strategy is to select suitable business projects according to external changes when integrating resources and creating products with core competitiveness. Enterprise strategy has the following characteristics: the research on strategic issues has the guiding significance of the overall situation, but the overall situation does not exist independently of the local. The focus of the strategy is by no means a year or a half, but a longer period. It should take the present as the starting point, look to the future, look at problems, and predict the future with new eyes. After a strategy is formulated, the difficulties encountered in its implementation are often more difficult than expected.

The management ability of an enterprise refers to the overall macrocontrol ability of an enterprise. The development planning of an enterprise requires a macrostrategic layout and planning. The ability of managers directly determines the realization of the long-term goal design of the enterprise. Whether an enterprise's strategic positioning can stand the test of the market depends primarily on the efficiency and effectiveness of the enterprise's management mechanism, as well as the sensitivity and execution of the market's response. This management system includes the improvement of the management system, the matching of the organizational structure, and the ability to integrate medium and long-term strategic planning and resources.

**Figure 2:**
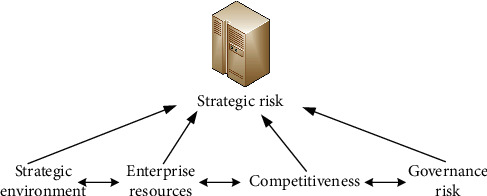
Enterprise risk factor framework model.

Enterprise strategic management refers to the positioning and selection of enterprise products and market competition areas according to changes in the environment. It is a dynamic management process that enables enterprises to achieve strategic goals in the analysis, evaluation, implementation, and control. The process of strategic management includes three factors: strategic task analysis, strategic task selection, and strategic goal implementation and control. The main purpose of strategic task analysis is to evaluate the key indicators that affect the strategic subject. By formulating strategic tasks, it points out the direction for the future of the enterprise and puts forward a practical development plan. Strategic task selection is mainly to point out where the future direction of the enterprise is. It focuses on the directional issues of research strategies, as well as the directional issues of enterprises in product research and development, market segmentation, and marketing strategy task selection. The results of a strategy take time to emerge, while the context in which a company executes its mission and plans is constantly changing. The key to strategy development lies in the right goals. The success or failure of the implementation of the strategy depends on whether the funds, personnel, technology, and other resources required for the implementation of the strategy can be effectively mobilized, and ultimately play an incentive role. After the analysis of the strategic task, there is the choice, and then the implementation. This seems to be in order, but in a specific work, not all companies have such a control process. The implementation of organizational strategy does not necessarily rely on this control process to achieve. As shown in Figure 2, it is an enterprise risk factor framework model.

The strategic risk is defined in the context of risk theory, especially in the fields of finance and futures. Strategic risk has always been seen as a detriment due to the inclination of national industrial policies. Strategic risk can be understood as the uncertainty of the enterprise as a whole. Among these losses, there are not only losses from the economic field but also losses from noneconomic fields, such as the decline of soft power, and so on. Among them, the loss suffered in the noneconomic field, the strategic risk defines it as the weakening of competitiveness, which is a loss of noneconomic benefits. To some extent, the specificity of strategic risk is not fully reflected. It defines strategic risk from the theory of decision-making system, emphasizing that strategic risk decision-making can bring overall risk, and its essence is also one of the forms of strategic risk. Starting from the goal of strategic management and control, certain inherent conditions ensure that the success of the strategy will not meet the reasonable needs at any time and have an adverse impact on the strategy. These concepts link the consequences of strategic risk to control objectives and are not sufficiently specific when taken together.

Through the analysis of the company's strategic management and control process, it is concluded that the company's strategic risk is obtained under the combined effect of strategic elements and risk response. These comprehensive factors constitute the normal operation of the enterprise's risk safety and are the necessary conditions for corporate risk management. When the conditions of these element clusters are not met, they are transformed into strategic risk factors.

The general characteristics of strategic risk are as follows: the impact of strategic risk is wider than that of general risk. This loss is inevitable for all risks. Therefore, when managing and controlling this risk, it is necessary to foresee losses in advance according to its characteristics and use means such as insurance, concealment, or transfer. Strategic risk is relatively more uncertain. The most essential characteristic of risk is its irreducibility. The goal of strategic risk management is to manage risks rather than eliminate potential risks. This is determined by the essential characteristics of risk. Risk management and control systems are not independent units. It is floating on top of the corporate organizational structure. Its essential characteristics are relevant to the activities of all organizations. Therefore, the organizational link of risk management and control is to fully intervene in risk management and control and establish an organized and innovative comprehensive strategic risk management and control system. Identifying the strategic risk of an enterprise should start from two aspects: one is the limitation of the enterprise's governance status and environmental resource factors, and the other is the influence of the individual's ability to understand and practice. If an enterprise has perfect technological innovation ability and organizational innovation ability, then compared with other enterprises, the enterprise can have the ability to grasp risk management and control in the overall situation.

### 2.2. Clustering Algorithm

The cluster analysis is derived from the taxonomy. In ancient classification theory, people relied heavily on professional experience and knowledge to achieve classification and rarely used mathematical tools for quantitative classification. As the humanities and technology evolved, the requirements for classification became higher, so accurate classification based on professional experience and knowledge is sometimes difficult. As a result, mathematical tools were gradually classified and formed a numerical classification. Subsequently, multivariate analysis technology for numerical classification was introduced to develop cluster analysis. Cluster analysis content is very rich, including systematic cluster method, sorted sample cluster method, dynamic cluster method, fuzzy cluster method, graphological cluster method, cluster prediction method, and so on.

The strategic environment should be analyzed from the macro- and microaspects of the enterprise. The macroenvironment is based on the overall situation, including political, economic, social, and other factors. The microenvironment is from the management of the details of the enterprise. It refers to a particular market sector environment in which an enterprise exists.

**Figure 3:**
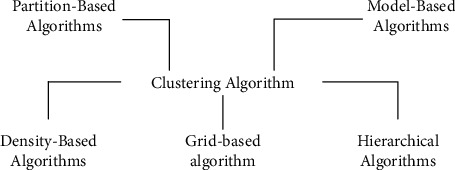
Classification of clustering algorithms.

The quality of clustering depends largely on the choice of similarity function. Choosing an appropriate similarity function is of great help to improve the clustering quality of the algorithm.  Figure 3 shows the classification of clustering algorithms.

Many clustering algorithms work very well in small databases with fewer than 200 data objects. However, a large database can contain several million parts. Grouping large samples of a data set can lead to biased results. Many cluster algorithms define clusters based on Euclidean or Manhattan distance measurements. Algorithms based on such distance measurements tend to find spherical clusters of the same size and density. However, the cluster can be of any shape. It is important to develop algorithms that can find clusters of any shape. Some cluster algorithms are sensitive to the order of the input data. For example, the same dataset, when presented with the same algorithm in a different order, can produce results from different clusters. It is important to develop algorithms that resist the sequence of data inputs.

Due to various reasons such as improper storage, most of the data in the real world will have incomplete data, inconsistent data types, abnormal noise interference, and so on. These data can be called dirty data. If these data are directly used for cluster analysis, it will seriously reduce the accuracy and efficiency of clustering. Therefore, data preprocessing is a very important part of cluster analysis. The four main data processing methods are the following: data cleaning: the main task of data cleaning is to standardize the format of data, remove unusual data and duplicate data, and correct data errors. The main operations are filling missing values, debugging noisy data, detecting outliers, etc. Data Integration: the main task of data integration is to combine and process heterogeneous data from different data sources and solve the problem of inconsistent data semantics. Data Transformation—the main task of data transformation is to transform data to make it suitable for data mining. The main operations include data smoothing, data aggregation, data aggregation, data normalization, etc. Data normalization: data normalization is an intermediate step performed on large datasets. The main purpose of data normalization is to remove some redundant data and rationalize it as much as possible without compromising data integrity. This mainly concerns the state of the data element and the state of the size, etc.

(1) $$\begin{array}{c} {\bar{m}}_{k}=\frac{1}{b}\sum_{u=1}^{b}M_{uk},\;k=1,2,\ldots ,K, \end{array}$$

where ${\bar{m}}_{k}$ is the average value of data object attributes.

(2) $$\begin{array}{c} D_{k}=\sqrt{\frac{1}{b-1}\sum_{u=1}^{b}{\left(m_{uk}-{\bar{m}}_{k}\right)}^{2}}, \end{array}$$

where *D*_*k*_ is the standard deviation of the attribute.

(3) $$\begin{array}{c} m_{uk}^{\prime }=\frac{m_{uk}-{\bar{m}}_{k}}{D_{k}},\;u=1,2,\ldots b, \end{array}$$

where *m*_*uk*_′ is the attribute standard deviation normalization.

(4) $$\begin{array}{c} E_{k}=\underset{1\leq u\leq b}{\text{max}}\left(m_{uk}\right)-\underset{1\leq u\leq b}{\text{min}}\left(m_{uk}\right), \end{array}$$

where *E*_*k*_ indicate extremely poor properties.

(5) $$\begin{array}{c} m_{uk}^{\prime }=\frac{m_{uk}-{\bar{m}}_{k}}{E_{k}},\;u=1,2,\ldots ,b, \end{array}$$

where *m*_*uk*_′ indicate attributes implement range standardization.

(6) $$\begin{array}{c} m_{uk}^{\prime }=\frac{m_{uk}-\underset{1\leq u\leq b}{\text{min}}\left(m_{uk}\right)}{E_{k}}, \end{array}$$

where *m*_*uk*_′ indicate attributes implement range normalization.

(7) $$\begin{array}{c} s\left(u,v\right)=\sqrt{\sum_{k=1}^{K}{\left(m_{uk}-m_{vk}\right)}^{w}},\;w>0, \end{array}$$

where *s*(*u*, *v*) is the Chebyshev distance.

(8) $$\begin{array}{c} \text{sin}\left(u,v\right)=\frac{\sum_{k=1}^{K}m_{uk}m_{vk}}{\sqrt{\sum_{k=1}^{K}m_{uk}^{2}\sqrt{\sum_{k=1}^{K}m_{vk}^{2}}}}, \end{array}$$

where sin(*u*, *v*) is the similarity of data objects.

(9) $$\begin{array}{c} l_{uv}=\frac{{\left[\psi \left(u,v\right)\right]}^{\alpha }\times {\left[\mu \left(u,v\right)\right]}^{\beta }}{\sum_{u=1}^{k}{\left[\psi \left(u,1\right)\right]}^{\alpha }\times {\left[\mu \left(u,1\right)\right]}^{\beta }}t_{\text{max}}, \end{array}$$

where *l*_*uv*_ is the probability that data objects belong to the same class and *α*, *β* is the regulator.

(10) $$\begin{array}{c} F=\text{min}\sum_{v=1}^{k}\sum_{m_{u}\in c_{v}}s\left(m_{u},a_{v}\right), \end{array}$$

where *F* is the sum of the dispersion within the class.

(11) $$\begin{array}{c} l_{l}\left(o_{u}\right)={\left(\frac{k_{1}}{k_{1}+f\left(o_{u}\right)}\right)}^{2}, \end{array}$$

where *l*_*l*_(*o*_*u*_) is the pickup probability and *k*_1_ indicate given parameters.

(12) $$\begin{array}{c} l_{s}\left(o_{u}\right)=\left\{\begin{array}{cc} 2f\left(o_{u}\right), & f\left(o_{u}\right)<k_{2},\\ 1, & \text{otherwise}, \end{array}\right. \end{array}$$

where *l*_*s*_(*o*_*u*_) is the drop probability and *k*_2_ indicate given parameters.

(13) $$\begin{array}{c} \varepsilon =\varepsilon_{0}\times \left(1-\frac{1}{\text{ceil}\left(t/t_{\text{min}}\right)+1}\right), \end{array}$$

where *ε*_0_ is the initial value and *ε* are adjustment parameters.

(14) $$\begin{array}{c} \text{sim}\left(u,v\right)=1-\sqrt{\frac{1}{A}\sum_{k=1}^{A}{\left(j_{uk}-j_{vk}\right)}^{2}}, \end{array}$$

where *u*, *v* are two data objects, *A* is the dimension of the data object, and *j*_*uk*_ is the attribute value of the data object.

(15) $$\begin{array}{c} W\left(m\right)=\sum_{u=1}^{b}L_{u}M_{u}, \end{array}$$

where *M*_*u*_ is the profit and loss value of the scheme in a certain state, *L*_*u*_ is the probability that a certain state will occur in the future and *b* is the number of future states.

(16) $$\begin{array}{c} \text{C}\cdot \text{E}=\frac{\text{C}\cdot \text{U}}{\text{E}\cdot \text{U}}<0.1,\\ f\left(s_{u}\right)=\sum_{v}y_{u}f\left(s_{uv}\right). \end{array}$$

where *f*(*s*_*u*_) is the evaluation value of the plan and C · E is the consistency condition.

(17) $$\begin{array}{c} e_{uv}=\frac{\sum_{k=1}^{a}\text{min}\left\{m_{uk},m_{vk}\right\}}{\sum_{k=1}^{a}\max\left\{m_{uk},m_{vk}\right\}},\\ M_{uv}^{\prime }=\frac{m_{uv}-\underset{u=1}{\wedge }m_{uv}}{\underset{u=1}{\vee }m_{uv}-\underset{u=1}{\wedge }m_{uv}}, \end{array}$$

where *M*_*uv*_′ is the data standardization for each indicator.

(18) $$\begin{array}{c} s_{uv}\left(w\right)={\left[\sum_{k=1}^{w}{\left|m_{uk}-m_{vk}\right|}^{2}\right]}^{1/2}, \end{array}$$

where *s*_*uv*_(*w*) is the Euclidean distance.

In a cluster analysis, for the same data set, using different clustering algorithms may produce completely different clustering results. Therefore, it is necessary to set a standard for measuring the quality of clustering results. In the cluster analysis algorithm, the quality evaluation of the clustering results is a very important stage. The process of evaluating the clustering results is the process of creating a scoring function according to the characteristics of the clusters. The function of the scoring function is to quantify the quality of the clustering results and give different scores according to the quality. But in practice, it is hard to have a scoring function that does this perfectly. At present, there are many kinds of evaluation functions, which can be mainly divided into three types: internal metric evaluation, external metric evaluation, and relative metric evaluation according to the different information used by the evaluation function.

**Figure 4:**
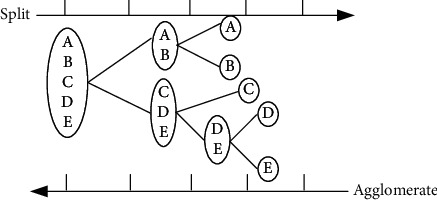
Schematic diagram of agglomeration and splitting.

Internal metric evaluation can be subdivided into cohesion metrics and separation metrics. Cohesion measures can reflect whether the internal data objects of a cluster are sufficiently correlated. The degree of separation measure can measure whether there is enough irrelevance between different families. The greater the correlation between the data objects within the cluster, the greater the irrelevance between the data objects between the clusters. The better the quality of the clustering, the higher the score of the evaluation function. In addition, the evaluation function also needs to consider the size of individual clusters to keep the evaluation balanced; otherwise, it is possible to generate trivial solutions that are worthless. In the extreme case, a data object is a cluster whose sum of squared errors is 0, but such a clustering is moot. A schematic diagram of agglomeration and splitting is shown in Figure 4.

Compared with internal metric evaluation, external metric evaluation is also called supervised measurement. External metric evaluation functions use external information, that is, information that is not present in the dataset. It is used to evaluate the degree of matching between the cluster structure and a specific external structure. When using an external metric evaluation function, the number of clusters in the dataset and the correct classification to which each data object belongs is known. Therefore, the external metric evaluation function can ignore the desired characteristics of the clusters. It does not need to consider whether the clustering results meet certain expectations but pays attention to the validity of the assignment and considers the matching degree between the class label and the actual label of the data object after the clustering is completed. The relative metric evaluation uses predefined evaluation criteria for the determined clustering algorithm, tests the algorithm by setting different parameters, and finally selects the optimal parameters and clustering mode.

## 3. Experiments and Analysis

**Figure 5:**
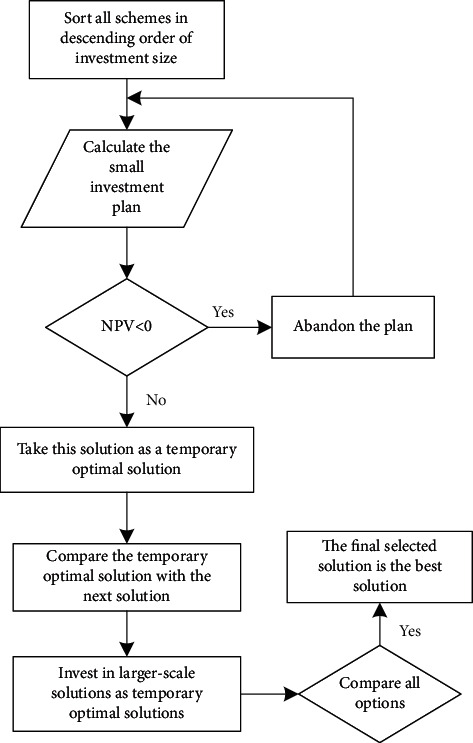
Delta analysis flowchart.

This paper conducts research on a company's strategic risk, builds a cluster analysis model, and analyzes corporate decision-making. Deterministic decision-making means that the conditions to be realized in the decision-making scheme are determined, and the result is also clear. Such decision-making problems are deterministic decision-making problems. The choice of mutually exclusive schemes is to choose one scheme over the other. In a way, it is a matter of merit.  Figure 5 shows the flow chart of the differential analysis method.

It lists all the combinations of independent programs and selects the project group whose investment amount is not greater than the capital budget. Each project group is treated as a new project. Then, according to the mutually exclusive scheme selection method, select the optimal project combination.  Table 1 shows the relevant data of the project. It can be seen from Table 1 that the combination of options B and C is the best, and the net present value is the largest, so it should be invested in B and C.

**Figure 6:**
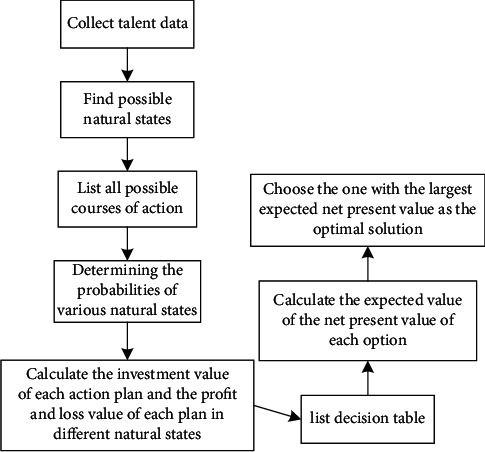
Decision-making steps of net present value and expected value method.

The expected value is a criterion for comparing the economic impact of different options. If the decision is based on how much profit, choose the option with the highest expected profit value. If the decision is cost-based, choose the option with the lowest expected cost. Expected value is the mathematical expectation of a discrete random variable, with probability. The sum of the gains and losses of the different possible scenarios and their respective probability multipliers is the expected value of the scenario. This approach requires decision-makers to determine the subjective probabilities of different natural states.  Figure 6 shows the decision-making steps of the net present value expected value method.

When judging the target, if there are many criteria factors that need to be considered, and these factors are in different positions, the criteria factors are usually analyzed and studied. They are divided into different levels to build a hierarchical model. For example, a developer intends to develop a new project and wants to understand the overall development environment in each region. In the preliminary investigation, in order to cooperate with the later fuzzy comprehensive evaluation, the content that needs to be understood is sorted and classified in advance, and a hierarchical structure model is established. The hierarchical model is shown in Table 2.

**Figure 7:**
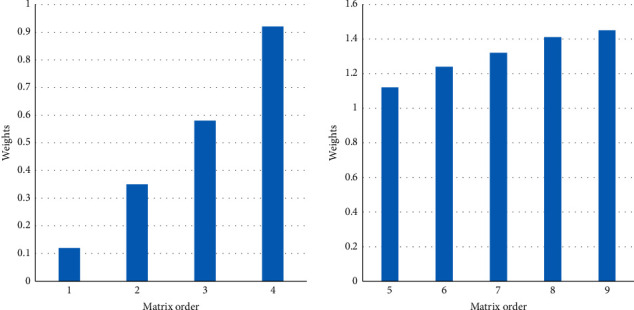
The value of the judgment matrix.

In the process of calculating the weight, each judgment matrix should be checked at one time to ensure the reasonableness of the obtained weight.  Figure 7 shows the value of the judgment matrix.

For each scenario, find the maximum value of all metrics for all scenarios. This maximum value is then subtracted from the value of each metric to get the standard ratio for each metric value.

These eigenvalues must have a clear practical relationship with the item under study, be highly discriminative, and be representative. A weighting method can be applied to the selected eigenvectors. This method assumes that the elements to be evaluated are divided into different levels according to their actual size. Criteria for each assessment are defined, and each element is assessed by experts or insiders according to its level.  Table 3 shows the weighted scoring method factor table.

**Figure 8:**
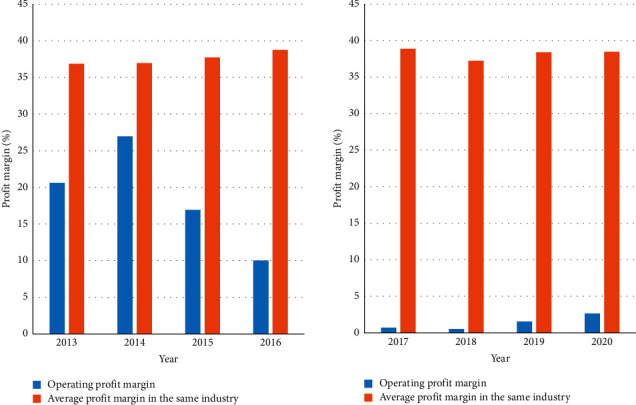
The company's operating profit margin and the industry's average operating profit margin.

It investigates the company's financial performance in recent years.  Figure 8 shows the company's operating profit margin and the industry average operating profit margin. It can be seen from the horizontal comparison that the operating profit rate of the company has been lower than that of the same industry. Although the profit margin in the first two years of diversification was almost the same as the industry average, with the gradual expansion of the diversification strategy, the company's operating profit margin dropped significantly, far below the industry average.

**Figure 9:**
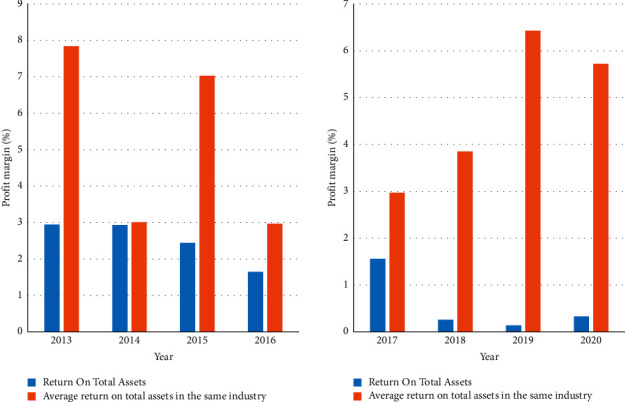
The company's return on total assets and the industry's average return on total assets.

Figure 9 shows the company's return on total assets and the industry's average return on total assets. It can be seen from the horizontal comparison that the company's current ratio is much higher than that of the same industry before it began to diversify its operations on a large scale. But after it began to gradually diversify, its current ratio plummeted to below the industry average. It can be seen from this that its short-term solvency defect and indicates that diversification has reduced the scale of the company's current assets.

Table 4 shows the company's net profit growth rate and the industry's average net profit growth rate. It can be seen that there has been an increase in these variables in recent years. However, due to the blind diversification of the company, even though it has no basis it insists on entering the business with huge capital demand, low correlation with the original core industry, and temporarily unclear prospects, making the capital chain unsustainable.

**Figure 10:**
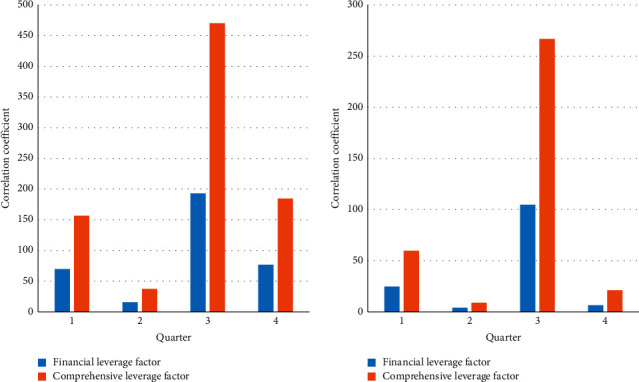
Risk leverage.

Based on the above data, a comprehensive risk analysis of the company is carried out, as shown in Figure 10, and this has the risk of leverage coefficient. It can be seen that the company's financial leverage has not fluctuated much in recent years, but from a numerical point of view, it is always in a high-risk state. Operating leverage and comprehensive leverage increased significantly and remained at a fairly high risk. This shows that the company has aggravated its financial and operating risks to a large extent while implementing a series of diversification behaviors, and the risks are far beyond the controllable range and can even affect the operation and development of the company.

The rapid expansion in many fields consumes huge amounts of capital, and the funds are seriously insufficient, which is highly reliant on bank loans. In the face of increased risks, banks tend to strengthen regulatory policies and tighten credit when capital flows dry up, leading to a full-blown risk. The financial risk management models and systems among the companies in this enterprise are very similar. The reason may be that the diversification strategy started with LeTV. In recent years, the rapid and diversified expansion of the seven major businesses has made it impossible for the management to quickly establish a management system suitable for enterprises in specific industries, so the company's risk management system is still used.

## 4. Discussion

When the operation of the existing business stops or for the purpose of supplementing the existing business, the enterprise implements diversified development, this means that the enterprise urgently needs new profit growth points. Therefore, when choosing a new business field, it should increase the depth of market research and analyze its own capabilities to find out potential new businesses in the future. It should first consider new businesses that are compatible with or complement the existing technical level and production capacity of the enterprise, so as to avoid possible risks and improve the success rate of diversified development. In order to avoid the risks of operating business, enterprises should adopt a related approach to implement diversified development. Practice has proved that the risk probability of related diversification is much lower than that of unrelated diversification. If there is a market supply and demand relationship in the industrial structure of the new business and the existing business, it will help the enterprise to control the market. If it is related to the operation mechanism, it will help to strengthen the internal control of the enterprise, further avoid possible problems and reduce the operation risk.

Since the enterprise has idle production capacity, in order to give full play to the idle resources, the enterprise implements diversified development and should fully evaluate the idle resources. If it can be marketed and utilized through the market, the enterprise does not need to diversify its operations to prevent the development of its main business from being affected. Companies that can only utilize resources through diversification should consider diversifying into new businesses. It first selects the associated diversity. The closer the correlation, the higher the utilization of the resource and the lower the risk. To sum up, diversified development is an important method for enterprise development and an integral part of the overall development deployment. Regardless of whether or not to develop diversification or to implement diversification for any purpose, how to develop diversification should focus on analyzing whether it helps to achieve the overall strategic purpose of the enterprise.

The implementation of enterprise strategy requires the coordination of corresponding environmental resources. As a necessary condition for the success of the strategy, if the abundance and availability of strategic resources are not met, it will turn into an uncertain risk factor. Enterprise strategic resources include technical resources, financial resources, market resources, and human resources. Technical resources refer to the industrial advantages formed by the research and development capabilities, technological innovation capabilities, and product market saturation of representative enterprises. These are the competitive advantages of enterprises. As a strategic entity, an enterprise must not only obtain valuable resources in market competition but also support innovation and meet strategic requirements. This is also the source of power for the development of enterprises and the country.

## 5. Conclusion

Both strategic decision-making and strategic control depend on the enterprise's manipulation of risk factors. Through the strategic analysis of the company, it is concluded that in order to prevent enterprise risks, the company divides the enterprise's strategic planning into three steps in the process of building a comprehensive industrial chain, namely strategy formulation, strategy implementation, and strategy adjustment. Every step is solid, and the enterprise introduces a risk factor at the strategic node of each step, effectively resisting the impact of internal and external adverse environments on the enterprise. Clustering algorithms have been introduced to enable more accurate predictions of corporate strategies and risks. This article is a preliminary study of the prophecies. Due to the limited data source and theoretical level, inaction in the study was inevitable. The analysis of the status assessment level is not detailed enough, it only shows the changes in the relevant indicators, and there is no internal assessment analysis. Due to the lack of mathematical knowledge, a suitable approach has not been found in the establishment and solution of the model. With the continuous development of computer linear programming software, the above problems will be solved.

## Figures and Tables

**Table 1:** Project related data.

Serial number	Scheme combination			Starting cost	Annual cash flow	Net present value	Internal rate of return (%)
	A	B	C				
1	1	0	0	10000	1698	432.11	12
2	0	1	0	6500	1300	1488.5	15.2
3	0	0	1	8500	1632	1528.64	14.05
4	0	1	1	15000	2932	3017.14	14.6

**Table 2:** Hierarchical models.

	First level	Second level
Hierarchical model	Political factors	Stable continuation of the policy
		Regional government support
	Economic factors	Industry market competitiveness
		Market effective demand
	Social factors	Perfection of public facilities
		Education level

**Table 3:** Weighted scoring method factor table.

Factor	Rating scale				Score
Environment	5	4	4	2	3
Transportation	4	4	3	2	2
Supporting facilities	5	5	4	1	4
Property management	3	4	4	2	3

**Table 4:** The company's net profit growth rate and the industry's average net profit growth rate.

Year	2015	2016	2017
Net profit growth rate	57.06%	87.5%	48.01%
Average net profit growth rate in the same industry	14.43%	11.69%	11.16%
Year	2018	2019	2020
Net profit growth rate	31.23%	42.57%	57.14%
Average net profit growth rate in the same industry	12.85%	11.42%	11.79%

## Data Availability

The data used to support the findings of this study are available from the author upon request.
